# Differential DNA methylation and lymphocyte proportions in a Costa Rican high longevity region

**DOI:** 10.1186/s13072-017-0128-2

**Published:** 2017-04-27

**Authors:** Lisa M. McEwen, Alexander M. Morin, Rachel D. Edgar, Julia L. MacIsaac, Meaghan J. Jones, William H. Dow, Luis Rosero-Bixby, Michael S. Kobor, David H. Rehkopf

**Affiliations:** 1 Department of Medical Genetics, Centre for Molecular Medicine and Therapeutics, BC Children’s Hospital Research Institute, University of British Columbia, 950 West 28th Ave, Vancouver, Canada; 2 School of Public Health, University of California, Berkeley, Berkeley, CA USA; 3 Centro Centroamericano de Población, Universidad de Costa Rica, San José, Costa Rica; 4 Division of General Medical Disciplines, Department of Medicine, School of Medicine, Stanford University, 1070 Arastradero Road, Suite 300, Palo Alto, CA 94304 USA

**Keywords:** DNA methylation, Epigenetics, Immune aging, Longevity, Biodemography, Epigenetic age

## Abstract

**Background:**

The Nicoya Peninsula in Costa Rica has one of the highest old-age life expectancies in the world, but the underlying biological mechanisms of this longevity are not well understood. As DNA methylation is hypothesized to be a component of biological aging, we focused on this malleable epigenetic mark to determine its association with current residence in Nicoya versus elsewhere in Costa Rica. Examining a population’s unique DNA methylation pattern allows us to differentiate hallmarks of longevity from individual stochastic variation. These differences may be characteristic of a combination of social, biological, and environmental contexts.

**Methods:**

In a cross-sectional subsample of the Costa Rican Longevity and Healthy Aging Study, we compared whole blood DNA methylation profiles of residents from Nicoya (*n* = 48) and non-Nicoya (other Costa Rican regions, *n* = 47) using the Infinium HumanMethylation450 microarray.

**Results:**

We observed a number of differences that may be markers of delayed aging, such as bioinformatically derived differential CD8+ T cell proportions. Additionally, both site- and region-specific analyses revealed DNA methylation patterns unique to Nicoyans. We also observed lower overall variability in DNA methylation in the Nicoyan population, another hallmark of younger biological age.

**Conclusions:**

Nicoyans represent an interesting group of individuals who may possess unique immune cell proportions as well as distinct differences in their epigenome, at the level of DNA methylation.

**Electronic supplementary material:**

The online version of this article (doi:10.1186/s13072-017-0128-2) contains supplementary material, which is available to authorized users.

## Background

Aging is a complex biological process that progressively leads to physiological decline and an increased risk of mortality. The genetic component of life span is approximated to be less than 30%, leaving the remainder to be determined by environmentally and socially influenced factors such as diet, exposure to infection, and lifestyle choices [1, 2]. While the mechanistic regulation of these non-genetic influences is poorly understood, previous work has suggested that epigenetic processes may be tightly interwoven with biological aging [3].

Epigenetics generally refers to the study of altered chromatin states, such as modifications to DNA and the proteins involved in its packaging and regulation. To date, DNA methylation (DNAm) is the most commonly studied epigenetic mark in human populations, as recent advances in technology have allowed for the inexpensive high-throughput measurement of >400,000 CpG sites across the genome. There are many other studied epigenetic processes, such as post-translational histone modifications, histone variants, and noncoding RNAs; however, these modifications have been more of a focus in model organism research and cancer biology.

DNAm is one type of epigenetic modification that impacts how genes are expressed and thus can have important phenotypic and functional consequences for an organism. Unlike the DNA sequence itself, DNAm is changeable through environmental influences over an individual’s life course. DNAm involves the covalent addition of a methyl group to the 5′ carbon of cytosine nucelotides, most often in the context of CpG dinucleotides (cytosine–phosphate–guanine). Genetic variants, such as single nucleotide polymorphisms (SNPs), can affect DNAm at nearby CpG sites, called methylation quantitative trait loci (mQTL) [4]. DNAm also varies in association with environmental and behavioral factors, such as diesel exposure and smoking. Additionally, variability in DNAm accumulates across one’s entire life span, discussed in detail below. DNAm patterns are associated with altered gene activity. Shifts in DNAm levels may follow a change in gene expression, or may act in the recruitment of methylation-dependent transcription factors that regulate transcriptional machinery. Understanding the effect of environmental influences on DNAm is important for unraveling the intricate regulation of genes and possible functional consequences of these alterations.

Age-related DNAm encompasses at least two distinct phenomena. First, specific CpGs associated with chronological age have been identified and, in some cases, replicated in several human populations. These age-related DNAm signatures can be either tissue specific or occur across several tissue types [5]. The epigenetic clock is a tool based on CpGs that change with age. Epigenetic clocks are DNAm-based markers of biological age, either confined to a single tissue or consistently accurate across tissues [6–8]. Deviations from these epigenetic age estimates, referred to as a measure of age acceleration, have been associated with an increased risk of all-cause mortality, time until death, and frailty [9–11]. Second, variability increases with age due to stochastic non-site-specific changes in DNAm, a process referred to as epigenetic drift [12].

It is critical to address cell-type heterogeneity when investigating DNAm patterns in tissues containing mixed cell types. Not only does cell type change over one’s life span but it is also the primary source of variation in DNAm across healthy individuals. DNAm profiles obtained from identical cell types, but separate individuals, show higher similarity than two different cell types from the same individual [13]. Given that isolating DNA from a single cell type is not always feasible or that cell count information is sometimes not available, bioinformatics-based methods have been developed to estimate cell-type proportions using DNAm profiles in blood and brain [14, 15]. The blood-based predictions are closely correlated with complete blood count measures, thus suggesting the validity of these methods to derive accurate blood count information bioinformatically [16]. It is also worth noting that measures of epigenetic age acceleration specific to whole blood have been defined to account for age-induced changes to cell-type proportions [6]. These measures are integral when analyzing DNAm from whole blood, as the proportion of certain blood cell types, such as CD8+ memory and naïve T cells, change with age [17].

Aging research in humans commonly investigates the unique biological and lifestyle characteristics of individuals surviving to old age [18]. An alternative approach, used in the current study design, is to examine the underlying biology of longevity by examining a population characterized as having a particularly high old-age life expectancy [19], the Nicoya region of Costa Rica, and comparing it to the rest of Costa Rica which has moderately lower life expectancy. By averaging out the stochastic variation in aging among individuals within each geographic region of the country, this approach offers a way to identify contextual (rather than individual) differences associated with healthy aging and longevity. While the method of examining area-based determinants of health and longevity has received substantial attention in biomedical research [20], a lack of appropriate data sources have limited its application in understanding the biological mechanisms of longevity.

The Nicoya peninsula of Costa Rica has been characterized by exceptionally high longevity, providing an intriguing framework to explore the relationship between DNAm and aging [21]. Mortality rates among elderly Costa Ricans in Nicoya are substantially lower than in the rest of Costa Rica, with individuals in Nicoya being some of the most long lived in the world. The relative mortality rate of Nicoya as compared to similar age cohorts in the rest of Costa Rica is 0.80. This advantage remains significant after statistical control for level of education and type of health insurance [21]. The Nicoyan advantage is particularly evident in cardiovascular disease, despite the fact that risk factors like smoking, physical activity and systolic blood pressure are similar throughout Costa Rica. One key indicator of the Nicoyan advantage is longer knee height—an anthropometric biomarker that is associated with early childhood environment [22]. Nicoyans also have lower BMI, waist circumference, and, among men, lower levels of HbA1c, glucose, triglycerides and total/HDL cholesterol ratio [21]. We have previously shown that leukocyte telomere length, an aging biomarker, also has more favorable (longer) levels among Nicoyans compared to individuals in the rest of Costa Rica [23].

In this study, we examined DNAm in a nationally representative population of Costa Ricans, investigating potential biological differences that may help explain the higher longevity observed in Nicoyans as compared to other Costa Ricans. We focused on DNAm, as this epigenetic mark can be modified by environmental influences, has the potential to regulate gene expression, and most importantly, has an established relationship with aging across the mammalian life span. Using genome-wide DNAm patterns to predict blood cell-type composition, we determined differential estimated proportions of age-related immune cells. While we did not observe differences in epigenetic age acceleration, we did find significantly decreased DNAm variability in Nicoyans. Finally, we identified DNAm patterns unique to Nicoyans, at both genomic regions and specific CpG sites. Understanding DNAm patterns between Nicoyans and other Costa Ricans (non-Nicoyans) will offer new insights both into the role of DNAm in aging and perhaps help to illuminate why Nicoyans have among the longest old-age life expectancies.

## Results

### Cohort characteristics and DNA methylation data

We examined a subset of samples from the Costa Rican Study on Longevity and Healthy Aging (CRELES), a longitudinal, nationally representative, and probabilistic sample of close to 3000 adults aged 60 years and over that were collected mostly in 2005, with over-sampling of older ages [24]. We assayed DNAm profiles of 48 Nicoyans (longevity group) and 47 non-Nicoyans (control group). In order to maximize statistical power for our age-based hypothesis, we randomly sampled half of the individuals between the ages of 60 and 75 and the other half aged 95 and above, selecting an equal number in each age category among Nicoyans and non-Nicoyans to have an age-matched sample. Table 1 shows the mean characteristics of these populations. Nicoyans tend to have lower levels of education and lower wealth than non-Nicoyans, but are similar on observed health-related characteristics. We obtained DNAm profiles from whole blood using the Infinium HumanMethylation450 (450k) array, a genome-wide microarray that quantifies DNAm at over 485,000 sites. We applied data quality controls to remove poor performing probes, probes that hybridized the XY chromosomes, and probes predicted to cross-hybridize [25]. Our final dataset for subsequent analyses consisted of 441,109 sites.

**Table 1:** Cohort characteristics (means and percents), Nicoyans and non-Nicoyans

Characteristics	Nicoya (*n* = 48)	Non-Nicoya (*n* = 47)
Age (mean in years)	83 (14)	85 (16)
Female (%)	57	55
Low education (%)	80	68
Low wealth (%)	35	21
Currently smoke (%)	4	6
Systolic blood pressure (mean mmHg)	139 (23)	140 (25)
Diastolic blood pressure (mean mmHg)	78 (12)	78 (13)
Body mass index (mean)	24 (7.1)	25 (5.8)

Standard deviations are shown in parenthesis. Low education is not completing primary school. The wealth index was based on a simple count of ten goods and conveniences in the household, ranging from running water and a toilet to having a cloth washer and a car. Low wealth is having six or fewer of these items. Systolic and diastolic blood pressure and body mass index were measured at the time of interview. Currently smoking was assessed through questionnaire. Further details on survey measures are available elsewhere [45]

### Nicoyans had fewer estimated CD8+ memory and more naïve T cells than non-Nicoyans

**Fig. 1:**
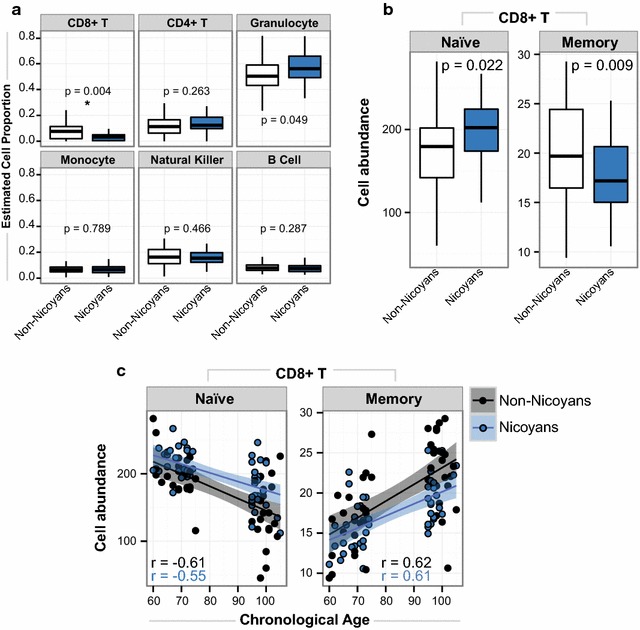
Nicoyans had differential CD8+ naïve and memory T cell abundance levels. **a** Box plots demonstrating bioinformatically derived white blood cells in Nicoyans and non-Nicoyans. Cell proportions estimated using the Houseman method.* Blue*: Nicoyans,* white*: non-Nicoyans. The *p* value is derived from a nonparametric group comparison test using Kruskal–Wallis. **b** Box plots illustrating the relationship between the bioinformatically derived CD8+ naïve T cell and CD8+ memory T cell across Nicoyans and non-Nicoyans. **c** Scatter plots of chronological age plotted against each CD8+ naïve T cells and CD8+ memory T cells abundance level for each sample. CD8+ naïve T cell show a decrease with age, whereas CD8+ memory T cells increase with chronological age. Pearson’s *r* coefficients derived from log-transformed age correlated with each respective cell-type level.* Blue*: Nicoyans,* black*: non-Nicoyans. Line of best fit shown with 95% confident intervals shaded in respective group color. The scale of cell abundance is a measure from a bioinformatically derived prediction of the respective cell types using flow-sorted counts from other datasets to infer cellular proportions of that specific isolated cell type based on the DNA methylation data [9, 46, 47]

Whole blood is a heterogeneous tissue containing certain cell types that change with age, with age-related decreases occurring in CD8+ T, CD4+ T and B lymphocytes, and the greatest increases in natural killer cells and monocytes [26]. To assess these differences in our cohort, we performed a previously described blood cell-type deconvolution [14] by using the DNAm profiles of each sample to estimate the proportions of granulocytes, natural killer cells, CD8+ T lymphocytes, CD4+ T lymphocytes, monocytes, and B lymphocytes. We found that Nicoyans had a significantly lower proportion of estimated CD8+ T cells when compared to non-Nicoyans (Kruskal–Wallis *p* value = 0.0038) (Fig. 1a). We also observed that Nicoyans had a higher mean level of estimated granulocyte proportions, although only reaching borderline significance (Kruskal–Wallis *p* value = 0.0486). It is important to note that we did not focus on the blood composition as a whole, as we were primarily interested in specific age-related cell-type trends.

To further investigate the differential proportion of estimated CD8+ T cells, we applied a more detailed cell deconvolution tool that provides an expanded estimation of CD8+ T naïve cells (CD8+ CD45RA+ CCR7+) and CD8+ T memory cells (CD8+ CD28− CD45RA−) [6]. CD8+ T memory cells typically increase with age, and CD8+ T naïve cells generally decrease with age through thymic involution [27]. Given that these measures are proportional and highly correlated, it is statistically appropriate to assess the ratio of CD8+ T naïve cells to CD8+ T memory cells (Additional file 1: Figure S1). Using this approach, we found a significant difference between Nicoyans and non-Nicoyans (Kruskal–Wallis *p* value = 0.0135). We observed a greater abundance of predicted CD8+ T naïve cells in Nicoyans and a lower abundance of estimated CD8+ T memory cells in Nicoyans, as compared to non-Nicoyans (Fig. 1b). These trends were suggestive of a younger immune cell profile in Nicoyans.

Pearson’s correlation coefficients were computed to assess the relationship between chronological age (log transformed) and the estimated proportion of each CD8+ T cell type (Fig. 1c). We observed a negative correlation between chronological age and CD8+ T naïve proportion (Nicoyans and non-Nicoyans; Pearson’s *r* = −0.55 and −0.61, respectively). As expected, there was a positive correlation between age and CD8+ T memory cells (Nicoyans and non-Nicoyans; Pearson’s *r* = 0.61, and 0.61, respectively; Fig. 1c), demonstrating a known immunological aging trend, but here based on epigenetic data. We found no significant difference in the regression slopes of chronological age on either CD8+ T cell type, when comparing Nicoyans to non-Nicoyans (*p* > 0.50); while we did observe differences in mean levels of both estimated CD8+ T cell types, we did not see any group difference in the nature of how these cell types changed across age.

### Epigenetic age did not differ between Nicoyans and non-Nicoyans

**Fig. 2:**
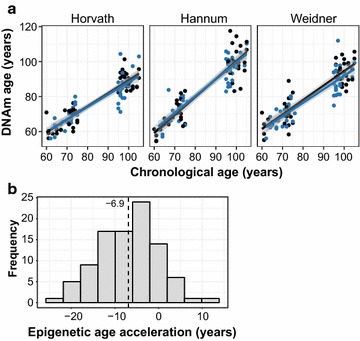
DNA methylation age correlated with chronological age. **a** *Left* to *right*: Horvath age estimation method was used to derive DNA methylation (DNAm) age using 353 CpGs; Hannum method to estimate DNAm age using 71 CpGs, and the Weidner 99 CpG model. **b** Histogram of age acceleration difference (DNAm age–chronological age). *Dotted line* shows the mean of all samples

Having established differences in estimated CD8+ T cell populations between the two groups, we next examined DNAm age as an established metric of biological aging. We examined DNAm age of Nicoyans compared to non-Nicoyans using three epigenetic clocks, which provided measures of biological age using DNAm levels at different groups of CpG sites (Fig. 2a) [6–8]. Across all samples, we found correlations between DNAm age and chronological age (Horvath: Pearson’s *r* = 0.86, Hannum: Pearson’s *r* = 0.85, Weidner: Pearson’s *r* = 0.86). However, we found no significant difference between Nicoyans and non-Nicoyans in terms of DNAm age (as calculated by each clock), while adjusting for chronological age (ANOVA *p* > 0.30, 95% CI for the Horvath clock was −6.3 to 3.8 years). We did, however, observe a mean difference of −6.9 years between epigenetic age and chronological age for all samples, suggesting that Costa Ricans, inclusive of both Nicoyans and non-Nicoyans, may on average be epigenetically younger than their chronological age (Fig. 2b). Furthermore, we reduced our data to only centenarians (age ≥ 100 years old), and the mean absolute difference between DNAm age and chronological age was −12.7 years.

We further examined biological age by assessing two other recently defined measures of age acceleration: intrinsic and extrinsic epigenetic acceleration, which are independent and dependent of blood cell-type proportions, respectively. We did not find any significant difference between Nicoyans and non-Nicoyans in any of these acceleration measures (ANOVA *p* ≥ 0.5, Additional file 2: Figure S2).

### DNA methylation variability was lower in Nicoyans

**Fig. 3:**
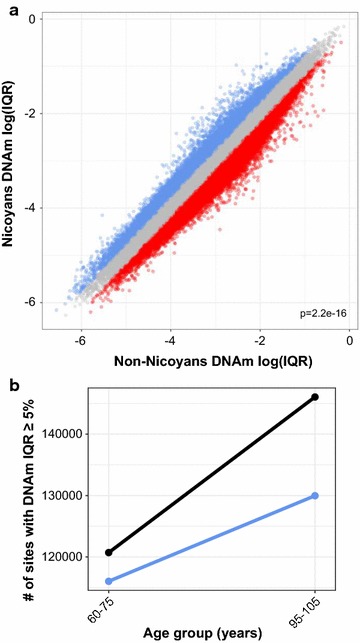
DNA methylation variability was lower in Nicoyans. **a** Scatter plot of log-transformed interquantile range (IQR; 90th–10th) at each CpG, values generated independently in each Nicoyan and non-Nicoyan group. *Blue* 41,695 CpGs had higher variability in Nicoyans. *Red* 98,073 CpGs had higher variability in non-Nicoyans. *Gray* insignificant variability, less than 20% IQR between groups. Significance value from Wilcoxon signed-rank test of IQR values between Nicoyans and non-Nicoyans. **b** Number of sites with a *β* value IQR greater than 5% in each age group. *Blue* Nicoyans, *gray* non-Nicoyans. *β* values were corrected for cell-type proportions

After assessing whether any differences in epigenetic age existed, we next investigated epigenetic drift between Nicoyans and non-Nicoyans. We hypothesized that lower variability in Nicoyans would be representative of a biologically younger profile based on the epigenetic drift phenomenon where stochastic variation in DNAm occurs with age. We calculated the interquantile range (IQR) at each CpG site (90th–10th percentile) represented on the 450k array to account for outliers and found a significant variability difference between Nicoyans and non-Nicoyans (Wilcoxon signed-rank test; *p* < 2.2 × 10^−16^), with a lower level of total mean DNAm variation in Nicoyans (Fig. 3a). Furthermore, we assessed the level of DNAm variability across individuals between 60 and 80 years old and individuals >80 years old, in Nicoyans and non-Nicoyans. We found a lower DNAm variability in the younger group, in both populations. However, there was a greater difference between the non-Nicoyan old and young age groups compared to the Nicoyans respective age groups (non-Nicoya: IQR mean difference = 0.0065, *p* < 2.2 × 10^−16^, Nicoya: IQR mean difference = 0.0043, *p* = 1.79 × 10^−10^). Lastly, when a *β* value IQR threshold of ≥5% was applied to only variable sites, we found 129,971 and 146,047 variable sites in the >80-year-old range of Nicoyans and non-Nicoyans, respectively. For the younger age range, we found 116,038 and 120,711 sites that had greater than 5% IQR in Nicoyans and non-Nicoyans, respectively (Fig. 3b). In summary, we found a lower degree of DNAm variability was associated with Nicoyans for both age groups, 60–80 years old and >80 years old, when compared to non-Nicoyans.

### Unique region-based differential methylation in Nicoyans

**Fig. 4:**
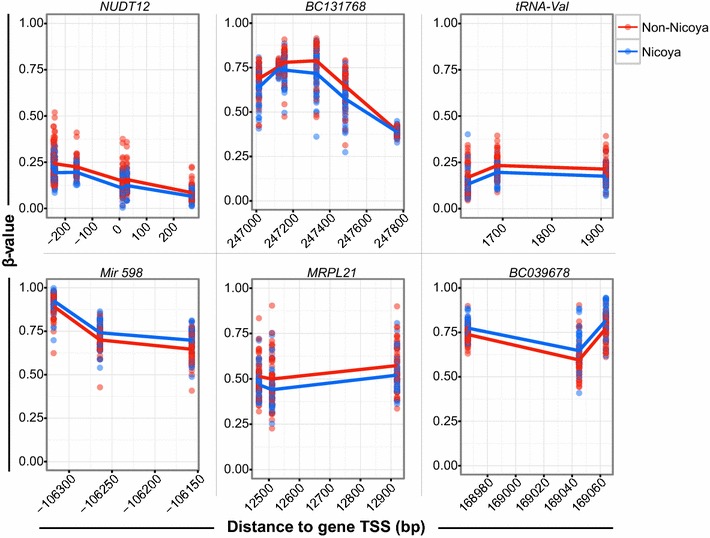
Significantly differentially methylated regions between Nicoyans and non-Nicoyans. Top six of 20 significant DMRs found using ‘DMRcate.’ Unadjusted *β* values are displayed on the *y*-axis and genomic distance (bp) to the most proximal gene transcriptional start site (TSS) is plotted on the *x*-axis. *Blue* Nicoyans, *red* non-Nicoyans. Group mean represented by respective *colored line*

We next investigated differentially methylated regions (DMRs) between Nicoyans and non-Nicoyans to identify unique epigenetic signatures that may be associated with the longevity observed in Nicoya. Using the R package ‘DMRcate,’ we found that in the comparison of Nicoyans and non-Nicoyans, 20 DMRs containing three or more CpG sites passed a false discovery rate of ≤0.05, as well as an arbitrary biological cutoff of a *β* value ≥ 5% between groups for at least one CpG site in each DMR (Table 2; Fig. 4; Additional file 3: Figure S3). Age, sex, and blood cell-type proportions were used as covariates. DMRs were associated with genes based on the closest proximity to a transcription start site (TSS). The mean length of the DMRs was 411 bp, with the shortest being 76 bp and the longest being 1416 bp. On average, out of the 20 DMRs observed, there were seven CpG sites per region, with a range from 3 to 16. Six DMRs were found within 1500 bp of a TSS associated with the following genes’ promoter region: Nudix Hydrolase 12 (*NUDT12*) (6 CpGs), Vault-RNA-2 (*VTRNA2*-*1*) (16 CpGs), Peptidase M20 Domain Containing 1 (*PM20D1*) (8 CpGs), Active BCR-Related (*ABR*) (3 CpGs), tRNA-Leu (5 CpGs), and LOC100128885 (uncharacterized) (3 CpGs). The majority of DMRs were found in intergenic regions (8/20) with an average number of four CpGs per DMR and average length of 267 bp. Four DMRs were found in the body (intragenic) of the following genes: Glutamate-rich protein 1 (*ERICH1*) (8 CpGs), Hook Microtubule Tethering Protein 2 (*HOOK2*) (4 CpGs), GATA2 Antisense RNA 1 (*GATA2*-*AS1*) (3 CpGs), and C15orf26 (uncharacterized) (4 CpGs). Two DMRs were found in the 3′end regions of Mitochondrial Ribosomal Protein L21 (*MRPL21*) (3 CpGs) and BolA family member 3 (*BOLA3*) (5 CpGs). The average absolute difference in DNAm between Nicoyans and non-Nicoyans was 7.9% when assessing the single CpG site for each DMR that showed the greatest difference between groups. When DNAm was averaged across the DMR per group, the mean *β* value difference was 5.9%.

**Table 2:** Differentially methylated genomic regions between Nicoyans and non-Nicoyans

Genomic sequence^a^	# CpGs	Min FDR	Max Δ*β*	Mean Δ*β*	Associated gene	DMR length (bp)	Genomic region	Gene ontology
chr5:102898223-102898733	6	1.0E−11	−0.07	−0.05	*NUDT12*	511	Promoter	NAD + diphosphatase activity; NADH pyrophosphatase activity
chr4:132896266-132897018	6	4.9E−08	−0.09	−0.06	–	753	Intergenic	–
chr5:180643432-180643713	3	1.9E−05	−0.05	−0.05	–	282	Intergenic	–
chr7:155832831-155832992	3	2.9E−04	0.06	0.05	–	162	Intergenic	–
chr11:68658383-68658836	3	7.3E−04	−0.09	−0.08	*MRPL21*	454	3′ end	Poly(A) RNA binding, ribosomal protein
chr6:6894084-6894182	3	5.3E−4	0.06	0.05	–	99	Intergenic	–
chr7:64034943-64035529	3	2.4E−03	0.07	0.06	*LOC100128885*	593	Promoter	–
chr12:130707332-130707407	3	2.6E−03	0.07	0.05	–	76	Intergenic	Wnt-protein binding
chr3:128215433-128216122	3	1.7E−03	−0.06	−0.05	*GATA2*-*AS1*	690	Intragenic	Enhancer sequence-specific binding, chromatin/transcription factor/C2H2 zinc finger binding
chr5:42924215-42924694	4	2.6E−03	0.07	0.05	–	480	Intergenic	–
chr1:152161237-152162507	8	2.1E−06	−0.07	−0.05	–	113	Intergenic	–
chr16:3988694-3988869	3	1.9E−02	−0.07	−0.05	–	176	Intergenic	–
chr6:28446794-28447115	5	1.3E−02	0.06	0.06	*tRNA*-*Leu*	322	Promoter	–
chr19:12876846-12877188	4	3.6E−02	−0.14	−0.11	*HOOK2*	343	Intragenic	Kinase modulator; membrane traffic protein
chr17:1133546-1133706	3	3.5E−02	−0.07	−0.05	*ABR*	161	Promoter	Transcription factor; protein binding; guanyl-nucleotide exchange factor
chr15:81410745-81411066	4	2.3E−02	0.09	0.06	*C15orf26*	322	Intragenic	–
chr2:74357527-74358223	5	2.0E−02	0.08	0.06	*BOLA3*	697	3′ end	Production of iron–sulfur clusters
chr1:205818956-205819609	8	2.2E−02	−0.08	−0.07	*PM20D1*	654	Promoter	Deacetylase, metalloprotease
chr8:599525-600940	8	2.7E−04	−0.12	−0.05	*ERICH1*	1416	Intragenic	–
chr5:135415693-135416613	16	1.1E−03	−0.09	−0.06	*VTRNA2*-*1*	921	Promoter	Potential tumor suppressor

*DMR* differentially methylated region

Δ*β*: (Delta beta; absolute mean or max difference of *β* values between groups (Nicoya–non-Nicoya)

^a^Genome coordinates from Human Genome GRCh37/hg19 Assembly

### Site-specific differential methylation in Nicoyans and technical verification

To complement the region-specific analysis, we also assessed DNAm differences at the site-by-site level to identify any single sites that were differentially methylated in Nicoyans as compared to non-Nicoyans. By investigating site-specific changes, we did not limit ourselves to highly represented genomic regions on the 450k array. We performed an epigenome-wide association study using a linear model of population group regressed on *M* values at each CpG site, with sex, age, and blood cell-type proportions included as covariates (Additional file 4: Figure S4). We observed nine CpGs below a nominal *p* value significance threshold of *p* ≤ 5 × 10^−7^ (*q* value < 0.022, Table 3) that were differentially methylated between Nicoyans and non-Nicoyans. After applying a biological threshold similar to the DMRcate analysis, four CpG sites passed our significance criteria: cg02853387 (*DSCAML1*), cg02438481 (*C6orf123*), cg13979274 (*OR10H1*), cg26107275 (*BC042649*). Not surprisingly, the four significant CpG sites did not overlap with our DMR findings, as these single CpGs identified through the site-by-site analysis were either: (1) found in a genomic region with no proximal array probes, or (2) nearby CpGs sites were not significantly correlated. The nearest neighboring array-CpG probe was greater than 1 kb away for three out of the four significant CpG sites (cg02853387, cg13979274, and cg26107275). While for cg02438481, the closest array-CpG (cg00788354) was 324 bp away, it had a Pearson’s correlation of *r* < 0.10 and was not significantly differentially methylated between groups.

**Table 3:** Characteristics of four biologically and statistically significant DNA methylation sites between Nicoyans and non-Nicoyans

Probe ID	Pyrosequencing		450k array			Chr	Distance to closest TSS (bp)	Genomic region: gene	Gene ontology
	Δ*β*	*p* value	Δ*β*	*p* value	*q* value				
cg02853387	0.06	7.9E−05	0.07	1.3E−07	0.012	11	183072	Intragenic: intron 3 of *DSCAML1*	Protein homodimerization activity
cg02438481	0.08	1.9E−05	0.08	1.6E−07	0.012	6	−1861	Intergenic: C6orf123	–
cg13979274	0.06	9.1E−07	0.06	2.0E−07	0.012	19	4347	Intergenic: ~ 3 kb from 3′ end of *OR10H1*	G-protein coupled receptor/olfactory receptor activity
cg26107275	–	–	−0.07	2.2E−07	0.012	12	−407	Promoter: *BC04264*	–

Δ*β*: (Delta beta; absolute mean difference of *β* values between groups (Nicoyans–non-Nicoyans)

*TSS* Transcription start site, *Chr* chromosome

**Fig. 5:**
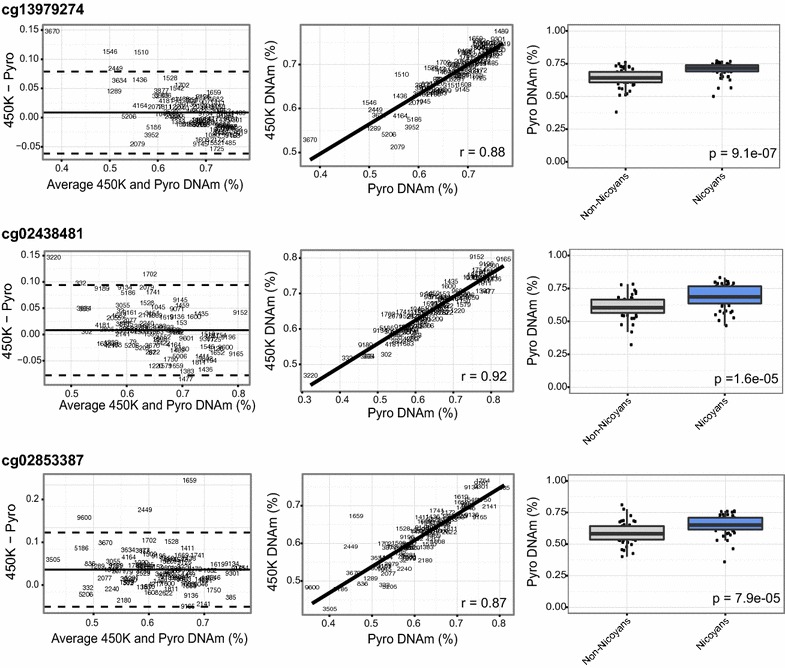
Pyrosequencing of significantly differentially methylated single CpGs. *Left* Bland–Altman plot of concordance between 450k array and pyrosequencing result for each CpG. *Text labels* represent sample IDs. *Middle* scatter plot displaying correlation between 450k array and pyrosequencing for each CpG. Spearman correlation coefficients shown. *Right* box plots of significant difference between Nicoyans and non-Nicoyans at each CpG site, measured using pyrosequencing. Significant value from regression model of CpG methylation on group status, while controlling for sex, age, and cell-type proportions

We performed pyrosequencing, a targeted DNAm sequencing technology, to verify that our single differential DNAm results were reproducible using an independent platform. We designed assays to measure three CpG sites that were observed to have a significant between group (Nicoyans and non-Nicoyans) difference in DNAm of ≥5.0% (cg02853387, cg02438481, and cg13979274) (sequences listed in Additional file 6: Table S1). Correlation coefficients between the pyrosequencing and 450k array for each CpG site showed a strong concordance between the two technologies [*r*_*s*_ = 0.87 (cg02853387), *r*_s_ = 0.92 (cg02438481), and *r*_s_ = 0.88 (cg13979274)]. Bland–Altman plots were generated for each CpG site to illustrate the agreement between the two quantification techniques (Fig. 5). To verify our differential DNAm findings between Nicoyans and non-Nicoyans, we confirmed these associations by statistically regressing DNAm determined by pyrosequencing onto group status, while controlling for age, cell-type proportions, and sex. All three sites remained significantly different between Nicoyans and non-Nicoyans (Table 3; Fig. 5).

Lastly, to determine whether a measure of genetic population structure was confounded with group (Nicoyans vs non-Nicoyans), we performed a post hoc analysis using ‘Epistructure’ [28]. Principal component analysis was completed on DNAm of CpGs previously identified as genetically informative loci. The first two principal components generated from this analysis have been proposed to confer composites of genetic structure to be used as covariates in a DNAm analysis. Using this technique, we did not observe a significant difference between the Epistructure principal components of the measures in Nicoyans and non-Nicoyans, while controlling for sex, age, and cell-type proportions (PC1: *p* = 0.60, PC2: *p* = 0.93, Additional file 5: Figure S5); therefore, we did not include these measures in our analyses.

## Discussion

In this study, we investigated differential patterns of DNAm in a population with well-characterized high longevity: Nicoya, Costa Rica. We aimed to identify unique patterns of DNAm that may underlie biological pathways associated with the longevity observed in Nicoya. Our study sample was drawn from a nationally representative demographic study of Costa Ricans age 60 years and over, and we randomly sampled individuals from within Nicoya and the rest of Costa Rica who were aged 60–75 and 95 years and above in order to assess age-associated DNAm. We have four primary findings. First, we observed a bioinformatically inferred younger immune profile in Nicoyan individuals compared to those living in the rest of Costa Rica, finding cellular proportion differences in CD8+ naïve and CD8+ memory T cells. Next, we found a lower level of total mean DNAm variation in Nicoyans compared to non-Nicoyans. We found 20 DMRs and four single CpG sites that were differentially methylated between Nicoyans and non-Nicoyans. While any of these differences we observed may be due to genetic differences in the populations, the fact that we show that estimated genetic structure did not differ between Nicoyans and non-Nicoyans suggests that these biological differences are more likely to be the result of environmental differences between these two populations. Lastly, DNAm age was not significantly different between Nicoyans and non-Nicoyans, although Costa Ricans overall had a younger mean DNAm age than the mean chronological age.

Our finding of proportional differences in CD8+ naïve and CD8+ memory T cells is intriguing in the context of previous work from animal models and other human research that has established that these blood cell proportions change as a function of age. Specifically, the naïve T cell response diminishes with time as they are naturally replaced by memory T cells through age-related thymic involution [17, 29]. Therefore, younger immune profiles have been hypothesized to delay the onset of infection vulnerability and extend health span [30]. Interestingly, centenarian offspring have been investigated in this context and show this ‘youthful’ immune cell phenotype [31]. The fact that our work suggested that Nicoyans had a lower proportion of CD8+ T memory cells and higher CD8+ T naïve cells is interesting in the context of immunoaging and might be suggestive of an age-related immune phenotype in Nicoyans.

Given their lower mortality rate, we were surprised about the lack of differences in DNAm age between Nicoyans non-Nicoyans, especially given that this biological aging measure has been associated with many age-related conditions such as cognitive fitness decline, frailty, and mortality [9, 11, 32]. It is important to note, however, that we only had the statistical power to test for a very large difference in DNAm age, and our 95% confidence interval suggests that Nicoyan individuals could be up to 6 years younger in DNAm age. We calculated epigenetic age in our samples using three published DNAm age predictors and found no significant difference in these measures of biological age between Nicoyans and non-Nicoyans. Previous work found that peripheral blood mononuclear cells from the offspring of semi-super centenarians, in an Italian cohort, appear 5.1 years epigenetically younger than controls. Centenarians also were reportedly 8.6 years younger than their chronological age [33]. This is consistent with our population; when we collapsed the Nicoyan and non-Nicoyan groups, we found centenarians were 12.7 years epigenetically younger. Furthermore, all Costa Ricans were 6.9 years epigenetically younger than their chronological age. It is possible that this might reflect the recently reported epigenetically younger phenotype in Hispanic populations [34].

To examine age-associated DNAm variability, we assessed a measure of variance in Nicoyans and non-Nicoyans independently. We were able to demonstrate the phenomenon of epigenetic drift within each of these groups, as our sample consisted of two age ranges in each group. Additionally, we observed that DNAm variation was lower in Nicoyans, both in the ≤80-year-old and in the >80-year-old age groups, when compared to non-Nicoyans. Given that DNAm variability across individuals has been reported to increase with age, our finding may highlight an epigenetic characteristic of the longevity in Nicoyans [12]. Although the biological pre- and antecedents of increased DNAm variability are poorly understood, our findings suggest lower DNAm variability may be associated with lower mortality. Some explanations for this age-associated DNAm variability have been proposed, such that DNAm variability may result from a functional decline in DNAm maintenance machinery or that the variability is a product of environmental exposures over time [35].

We found 20 genomic regions and four single CpGs that were significantly differentially methylated between Nicoyans and non-Nicoyans. One such DMR contained six CpGs and was located in the promoter region of *NUDT12*, a gene encoding a protein shown in vitro to cleave NADH, NADPH, and NAD+ [36]. Given that NUDT12 may play a role in NAD metabolism, a regulatory process associated with health span and aging, it is consistent with the fact that DNAm may be involved in the regulation of this gene that may have downstream effects on NAD biosynthesis. Nicoyans had a lower level of DNAm in the promoter region of *NUDT12*, a signature often associated with higher gene expression. In addition to our DMR findings, we also found four individual sites to be both statistically and biologically significant, three of which existed in intergenic regions. We further investigated three of these CpGs by quantifying DNAm with pyrosequencing, allowing us to verify both the accuracy of the 450k array and the significant differences between Nicoyans and non-Nicoyans. However, it remains unclear whether differential DNAm of these single CpGs or DMRs, at the observed effect sizes (<10%), are sufficient to yield a biological change. Interpretation of these findings at a biological level will require future mechanistic experiments.

Our findings should be interpreted within the context of several limitations. One limitation was the lack of genotype information for these samples, as genetic variation is considerably associated with DNAm [37]. In order to reduce genetic heterogeneity, we restricted control sampling to areas within Costa Rica, but outside of Nicoya. Nicoyan status is determined as of the time of the survey, i.e., at older ages, not based on birth or life-course residence, but in our sample 44 out of 48 Nicoyan residents have lived there their entire lives. While there are no documented differences between the historical migration patterns of the inhabitants of Nicoya and the rest of Costa Rica, minor differences may exist. Therefore, we implemented a recently published tool to infer genetic information using DNAm data obtained from the 450k array called ‘Epistructure,’ a tool from the python package GLINT [28]. We found no significant difference in population structure measures between Nicoyans and non-Nicoyans and thus did not include these composite measures in our analyses. Another consideration of our study results is that we used a bioinformatics approach to predict CD8+ T cell proportions, which are relative compositional estimates. As these predictions do not estimate actual cell counts, the abundance of other cell types will affect the proportional estimate of the CD8+ T cells. Ideally, we will need to validate our findings using a quantitative approach, such as fluorescent-activated cell sorting, to obtain actual cell counts. We note, perhaps not surprisingly, that we did not observe a significant difference when we performed an overall compositional analysis of these predictions [38], meaning that the blood composition overall was not different between Nicoyans and non-Nicoyans. However, our findings are supported by using two separate reference-based approaches [6, 14], both of which identified CD8+ T cells as being significantly different between Nicoyans and non-Nicoyans. Furthermore, these bioinformatic cell-type proportion predictions have been well validated in the literature when compared to actual cell counts, and so we were confident that this approach reflected true cell-type proportions [16].

## Conclusions

Our findings thus highlight DNAm as a potential factor underlying the unique longevity observed in Nicoya region of Costa Rica. This work also supports the demographic data on longevity as characterizing this population as unique. The specific differences in immune cell proportions we observed in Nicoyans will lay the framework for a validation study to observe whether cell-sorting experiments yield similar results. Additionally, the differential DNAm findings may provide a candidate list of CpGs to test for differences in other longevity populations. Lastly, upon validating our findings, our work will contribute to narrowing the focus of mechanistic studies to assess whether the DNAm differences we observed are involved in gene regulation that may alter gene expression trajectories.

## Methods

### Sample preparation and data collection

Whole blood was collected from participants and genomic DNA was extracted at the University of Costa Rica from 2 ml of frozen whole blood using the phenol–chloroform method. DNA was bisulfite converted with the Zymo Research EZ DNA Methylation™ Kit (Irvine, CA, USA). Approximately 160 ng of bisulfite-converted DNA from each sample, with the addition of one technical replicate, was randomized across eight 450k array BeadChips as well as sentrix row and run in one batch according to the manufacturer’s protocol (San Diego, CA, USA).

Qiagen Pyromark Assay Design 2.0 software (Hilden, Germany) was used to generate pyrosequencing assays targeted to three 450k array CpGs. Pyrosequencing was performed on the a Qiagen Pyromark™ Q96 (Hilden, Germany) according to manufacturer’s instructions. All primer sequences are listed in Additional file 6: Table S1.

### Data preprocessing and normalization

Illumina GenomeStudio software (San Diego, CA, USA) was used to subtract background noise and color correct raw data using control probes. Data were extracted in the form of an average *β* value matrix and imported into R Statistical software for the remainder of data processing. Logit-transformed *β* values to *M* values, a less heteroscedastic value, were used for all statistical analyses, whereas *β* values were used for visualization purposes as they represent percent methylation (0 = no methylation, 1 = fully methylated). We have included a comparison table of *β* values compared to *M* values for CpGs identified in the site-specific differential methylation analysis (Additional file 7: Table S2).

All data processing and statistical analyses were implemented in R version 3.2.3. We removed a subset of probes that could potentially lead to erroneous results. These consisted of 65 SNP control probes, probes that were specific to either the X or Y sex chromosomes, probes with missing *β* values or with a detection *p* ≥ 0.01 in 5 or more samples, polymorphic CpG probes, and cross-hybridizing XY probes. The total number of probes post-filtering based on these criteria was 441,109 [25]. No sample outliers were removed, defined as having more than 5% of their total probes fail.

Subset-quantile within array normalization (SWAN) was used to account for type I and II probe differences on the 450k array [39]. Known technical variation (sentrix ID and position) was accounted for with the function ‘ComBat’ [40]. Confirmation of these corrections was assessed before and after using principal component analysis.

### Estimation of blood cell proportions

A validated cellular deconvolution method was used to estimate cell-type proportions in each blood sample, namely CD4+ and CD8+ T cells, natural killer cells, B cells, monocytes and granulocytes [16]. The predicted abundance levels of CD8+ T naïve and CD8+ T memory were obtained from the ‘Advanced Blood Analysis’ of the online DNAm age predictor [6]. Significance values were generated from performing a Kruskal–Wallis test for each cell-type proportion by group.

### Prediction of epigenetic age

Three epigenetic clocks were used to predict biological age. The ‘Horvath’ and ‘Hannum’ estimates were computed with the online epigenetic clock software [6, 7]. The ‘Weidner’ age prediction was generated using the previously described 99 CpG model [41]. We investigated, to the best of our ability, the possibility that this finding was due to a global batch effect influencing all samples by performing the DNAm age calculation on raw data, after SWAN normalization data, and again after ComBat correction. In all cases, the mean DNAm age for all Costa Ricans was younger than the mean chronological age. We chose to proceed with calculating DNAm age using the most corrected data as we expected data that is corrected for technical batch effects, inclusive of probe design and chip–chip variance, to best represent true biological signal.

### DNA methylation analysis

The R package ‘DMRcate’ was used to find DMRs [42]. The DMRcate model contained Nicoya group, chronological age, sex, and estimated cell-type proportions. This tool uses a Gaussian kernel smoothing of DNAm across the genome. Benjamini–Hochberg (BH) method was applied with a threshold of ≤ 0.05 and a *β* value difference of ≥ 5% [43].

Site-specific differential DNAm analysis was conducted using moderated t-statistics with empirical Bayesian variation estimation using the bioconductor R package ‘limma’ with chronological age, sex, and cell-type proportions as covariates [44]. *M* values consisted of log-transformed *β* values to achieve a measure with uniform variation and decreased heteroscedasticity. Significance values were corrected for multiple testing using the BH method [43].

DNAm variability was calculated using the interquantile range (IQR) across the 90th and 10th percentiles of each group, independently, at each CpG. A significance value was generated by performing a Wilcoxon signed-rank test between groups.

### Inferred genetic ancestry

Population structure was inferred using the ‘Epistructure’ command-line tool GLINT [28]. This method applies principal component analysis on a reference list of genetically informative 450k array probes. This tool suggests the top two principal components can infer genetic structure. Linear regression was used for comparison of each PC and group status, while adjusting for sex, cell-type proportions, and age.

## Additional files

**Additional file 1: Figure S1.** Correlation plot of DNA methylation-based estimated blood cell-type proportions. Colored blocks represent correlation p values below 0.05, red indicates negative correlation and blue indicates positive correlation. Gran = granulocyte, Mono = monocyte, NK = natural killer.

**Additional file 2: Figure S2.** EEAA (extrinsic epigenetic age acceleration), General Age Acceleration (residuals from a linear model of DNAm age regressed onto chronological age), IEAA (intrinsic age acceleration). All measures were generated from the online epigenetic age software. No significant differences were observed between Nicoyans (blue) and non-Nicoyans (red). Significant values generated from ANOVA statistical tests.

**Additional file 3: Figure S3.** Continuation of differentially methylated regions between Nicoyans and non-Nicoyans. Remaining 14 of the 20 statistically significant DMRs obtained from DMRcate analysis found by the R package ‘DMRcate.’ Unadjusted DNA methylation values, shown as percent of cells methylated, are displayed on the y-axis, and genomic distance (bp) to the TSS is plotted on the × axis. Associated genes are based on closest distance to the TSS of each differentially methylation region. Nicoyans are represented by blue points with the mean of each CpG site illustrated by a blue line. Non-Nicoyans are represented with red with each point indicating an individual, with the red line illustrating the mean at each CpG.

**Additional file 4: Figure S4.** QQ plots of each identified CpG modeled using M values or β values. Linear model included DNA methylation value regressed on group (Nicoya vs non-Nicoya) with sex, age, and estimated cell-type proportions included as covariates.

**Additional file 5: Figure S5.** Epistructure-derived principal component analysis. Genetically informative 450k array sites were used to estimate genetic population structure in our data. Principal component analysis was performed on a reference CpG list to generate measures (PCs) of genetic structure. No significant difference in these estimates was seen between Nicoyans and non-Nicoyans.

**Additional file 6: Table S1.** Pyrosequencing primer sequences designed with Qiagen Pyromark Assay Design 2.0 software.

**Additional file 7: Table S2.** Comparison of M values and β values of each identified CpG.

## Abbreviations

450k array: Infinium HumanMethylation450 array
BH: Benjamini and Hochberg
CpG: cytosine–phosphate–guanine dinucleotide
DMR: differentially methylated region
DNAm: DNA methylation
FDR: false discovery rate
IQR: interquantile range
PCA: principal component analysis
