# Supplementary figures and images for: Differential DNA methylation and lymphocyte proportions in a Costa Rican high longevity region
Source: Epigenetics Chromatin. 2017 Apr 27;10:21. doi: 10.1186/s13072-017-0128-2 (PMC5408416; doi:10.1186/s13072-017-0128-2)

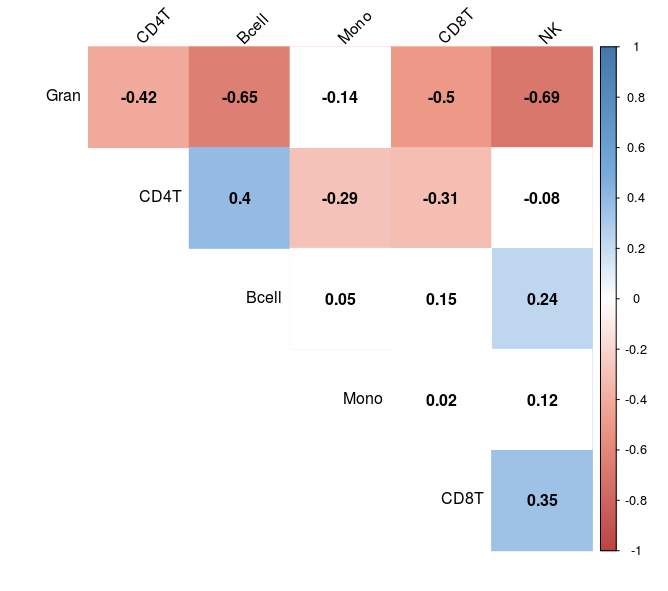

Supplement: Supplementary file 1 — Additional file 1: Figure S1. Correlation plot of DNA methylation-based estimated blood cell-type proportions. Colored blocks represent correlation p values below 0.05, red indicates negative correlation and blue indicates positive correlation. Gran = granulocyte, Mono = monocyte, NK = natural killer. [file 13072_2017_128_MOESM1_ESM.png]

Age Acceleration

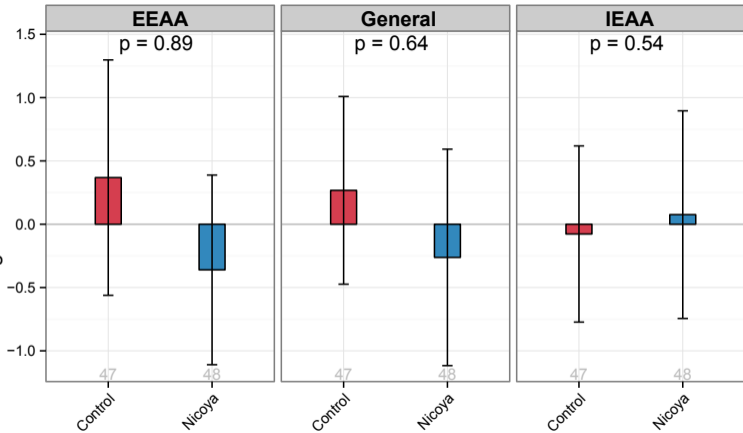

Supplement: Supplementary file 2 — Additional file 2: Figure S2. EEAA (extrinsic epigenetic age acceleration), General Age Acceleration (residuals from a linear model of DNAm age regressed onto chronological age), IEAA (intrinsic age acceleration). All measures were generated from the online epigenetic age software. No significant differences were observed between Nicoyans (blue) and non-Nicoyans (red). Significant values generated from ANOVA statistical tests. [file 13072_2017_128_MOESM2_ESM.pdf]

Beta Value

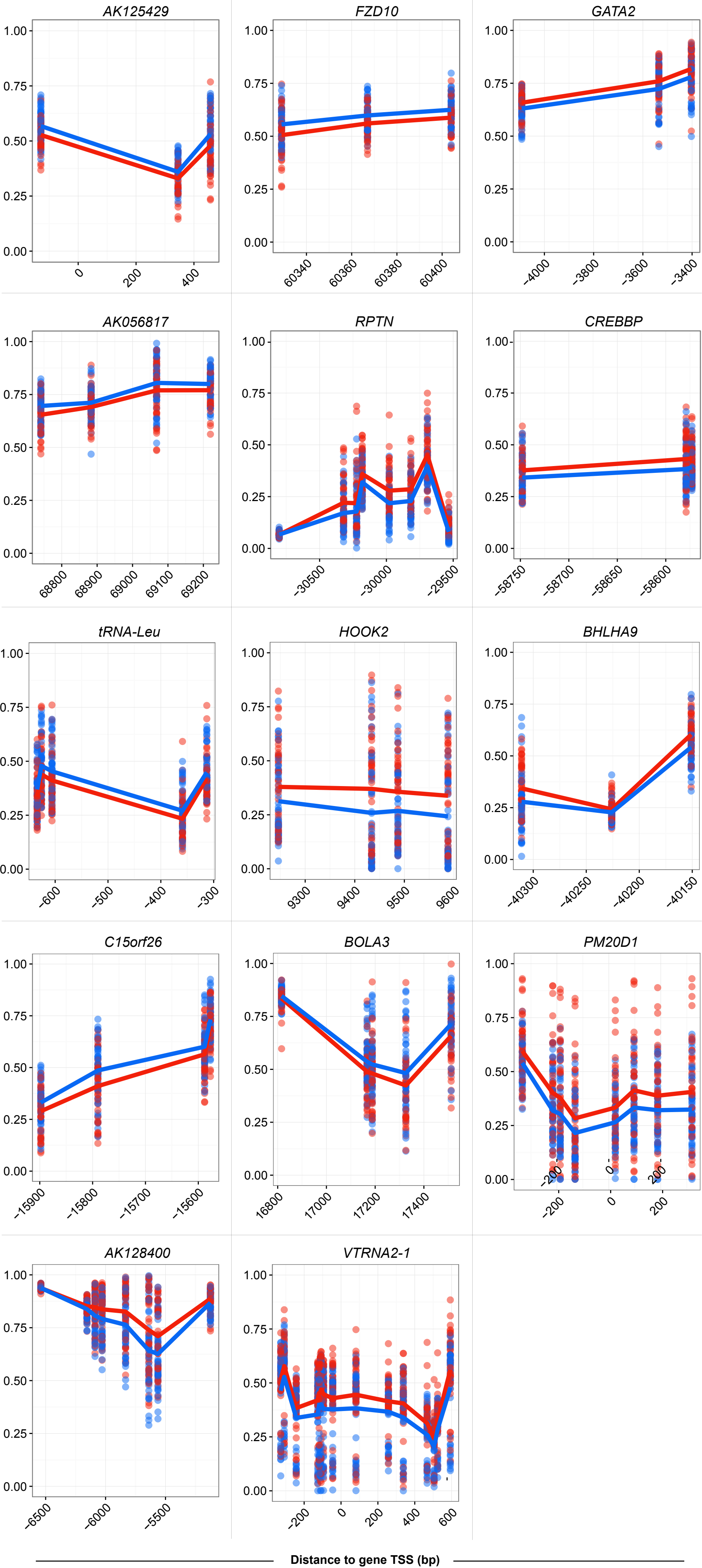

Supplement: Supplementary file 3 — Additional file 3: Figure S3. Continuation of differentially methylated regions between Nicoyans and non-Nicoyans. Remaining 14 of the 20 statistically significant DMRs obtained from DMRcate analysis found by the R package ‘DMRcate.’ Unadjusted DNA methylation values, shown as percent of cells methylated, are displayed on the y-axis, and genomic distance (bp) to the TSS is plotted on the × axis. Associated genes are based on closest distance to the TSS of each differentially methylation region. Nicoyans are represented by blue points with the mean of each CpG site illustrated by a blue line. Non-Nicoyans are represented with red with each point indicating an individual, with the red line illustrating the mean at each CpG. [file 13072_2017_128_MOESM3_ESM.pdf]

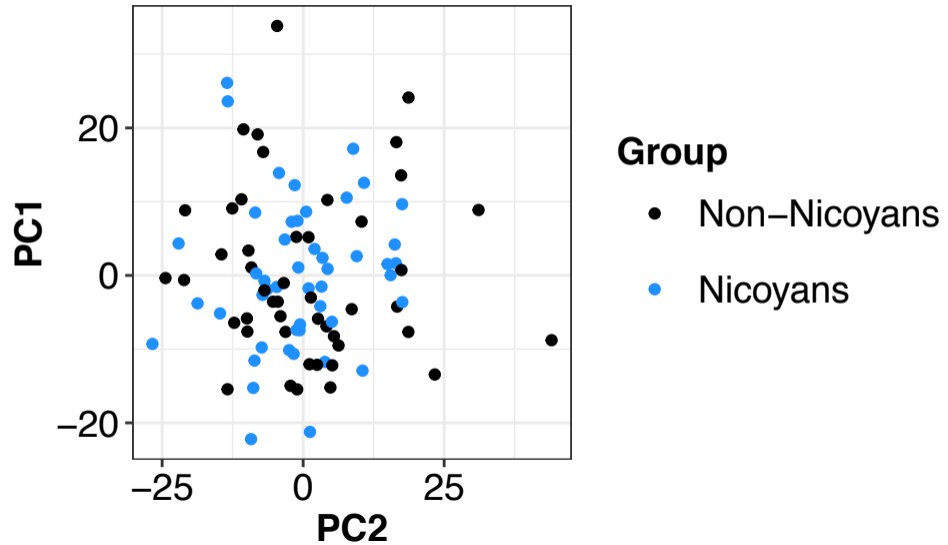

Supplement: Supplementary file 5 — Additional file 5: Figure S5. Epistructure-derived principal component analysis. Genetically informative 450k array sites were used to estimate genetic population structure in our data. Principal component analysis was performed on a reference CpG list to generate measures (PCs) of genetic structure. No significant difference in these estimates was seen between Nicoyans and non-Nicoyans. [file 13072_2017_128_MOESM5_ESM.pdf]
