# Supplementary material for: Differential DNA methylation and lymphocyte proportions in a Costa Rican high longevity region
Source: Epigenetics Chromatin. 2017 Apr 27;10:21. doi: 10.1186/s13072-017-0128-2 (PMC5408416; doi:10.1186/s13072-017-0128-2)
Supplement: Supplementary file 4 — Additional file 4: Figure S4. QQ plots of each identified CpG modeled using M values or β values. Linear model included DNA methylation value regressed on group (Nicoya vs non-Nicoya) with sex, age, and estimated cell-type proportions included as covariates. [file 13072_2017_128_MOESM4_ESM.pdf]

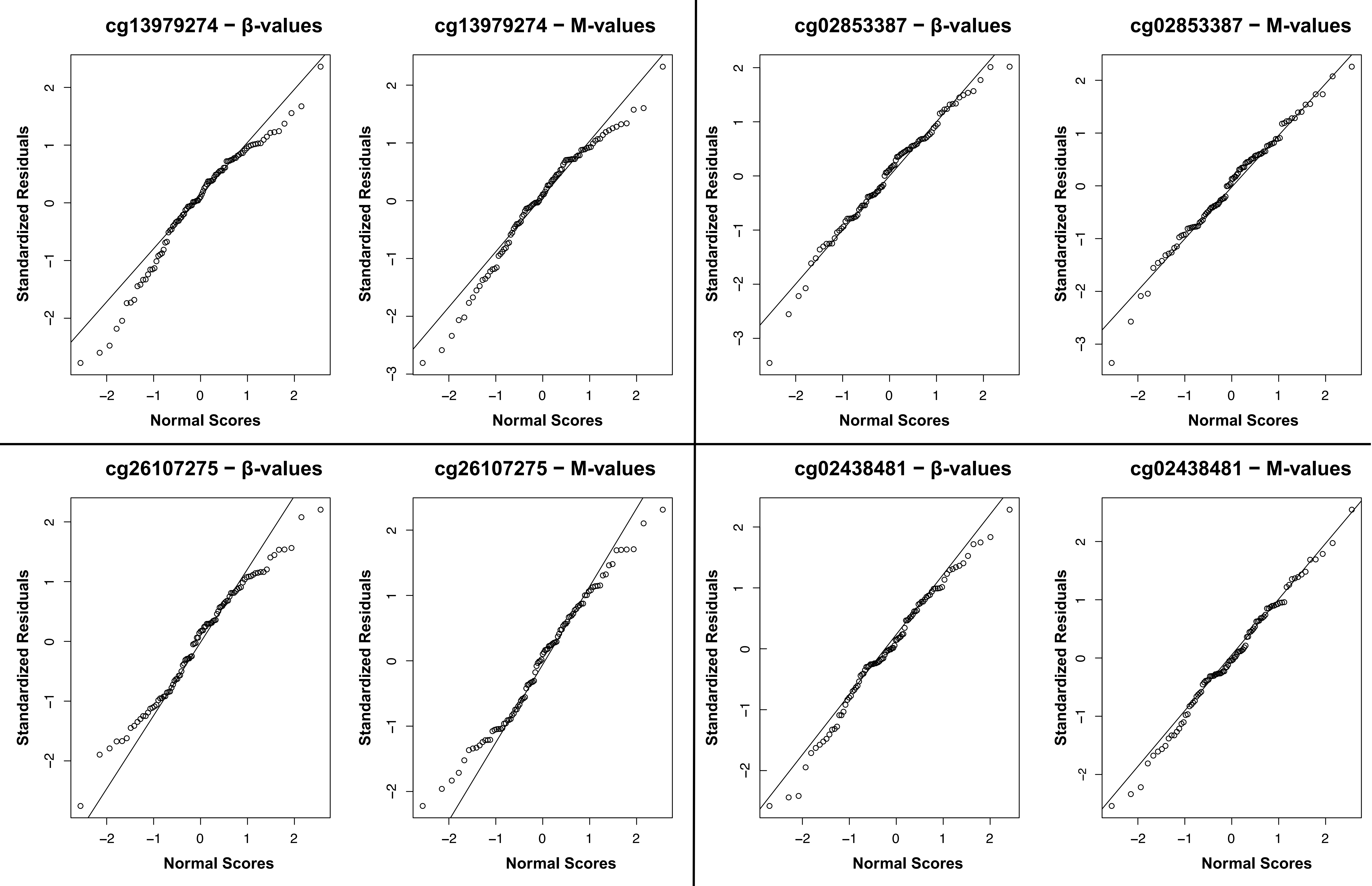
cg13979274 − β-values
cg13979274 − M-values
cg02853387 − β-values
cg02853387 − M-values
cg26107275 − β-values
cg26107275 − M-values
cg02438481 − β-values
cg02438481 − M-values
Standardized Residuals
Normal Scores
