# Supplementary material for: Differential DNA methylation and lymphocyte proportions in a Costa Rican high longevity region
Source: Epigenetics Chromatin. 2017 Apr 27;10:21. doi: 10.1186/s13072-017-0128-2 (PMC5408416; doi:10.1186/s13072-017-0128-2)
Supplement: Supplementary file 6 — Additional file 6: Table S1. Pyrosequencing primer sequences designed with Qiagen Pyromark Assay Design 2.0 software. [file 13072_2017_128_MOESM6_ESM.pdf]

**Additional file 6: Table S1.** Pyrosequencing primer sequences designed with Qiagen Pyromark Assay Design 2.0 software.

| Assay CpG target | Primer | Sequence |
|---|---|---|
| cg02853387 | Forward | AGGGAAGAAAAGTTATTAAGTTGT |
| | Reverse (5'biotinylated) | ACAAATACAAAACCCATATTCTCAA |
| | Sequencing | GTGTAGGTTTTTAGTTTATAGT |
| cg02438481 | Forward | GTTTTGGGTTTGGTGATTTGGTTTTA |
| | Reverse (5'biotinylated) | ATTTCTTAATCAATACCACCTTCTTCTATA |
| | Sequencing | TTTATTTTAGGTGGGAGT |
| cg13979274 | Forward (5'biotinylated) | AGGGGAGTATTTTAGTTTAGTGTATAG |
| | Reverse | CCAACTTAAAAAAACCAAACTTCAATATC |
| | Sequencing | AAAACAATTACAACCCTC |
