# Supplementary material for: Differential DNA methylation and lymphocyte proportions in a Costa Rican high longevity region
Source: Epigenetics Chromatin. 2017 Apr 27;10:21. doi: 10.1186/s13072-017-0128-2 (PMC5408416; doi:10.1186/s13072-017-0128-2)
Supplement: Supplementary file 7 — Additional file 7: Table S2. Comparison of M values and β values of each identified CpG. [file 13072_2017_128_MOESM7_ESM.pdf]

Additional file 7. Table S2. Comparison of M-values and β-values of each identified CpG.

CpG ID	Performed on M-values		Performed on beta values	
	p-value	q-value (BH corrected)	p-value	q-value (BH corrected)
cg02853387	1.3E-07	0.012	1.6e-07	0.014
cg02438481	1.6E-07	0.012	2.2e-07	0.016
cg13979274	2.0E-07	0.012	2.9e-07	0.018
cg26107275	2.2E-07	0.012	3.2e-07	0.018
